# P-1116. Distribution of Rifaquizinone in Bone, Synovial Fluid and Plasma: A Phase 1 Trial in Patients Undergoing Total Hip or Knee Arthroplasty

**DOI:** 10.1093/ofid/ofae631.1303

**Published:** 2025-01-29

**Authors:** Javad Parvizi, Chad A Krueger, Jing Chen, Huan Wang, Changlin Ai, Guozhu Geng, Zhenkun Ma

**Affiliations:** Rothman Orthopaedic Institute, Philadelphia, Pennsylvania; Rothman Orthopaedic Institute, Philadelphia, Pennsylvania; TenNor Therapeutics (Suzhou) Ltd, Suzhou, Jiangsu, China (People's Republic); TenNor Therapeutics (Suzhou) Ltd, Suzhou, Jiangsu, China (People's Republic); TenNor Therapeutics (Suzhou) Ltd, Suzhou, Jiangsu, China (People's Republic); TenNor Therapeutics (Suzhou) Ltd, Suzhou, Jiangsu, China (People's Republic); TenNor Therapeutics, Suzhou Industrial Park, Jiangsu, China (People's Republic)

## Abstract

**Background:**

Rifaquizinone (RFQ, TNP-2092) is a novel multitargeting drug conjugate in development for the treatment of prosthetic joint infections (PJIs). RFQ exerts its antibacterial activity by inhibiting RNA polymerase, DNA gyrase, and topoisomerase IV. This study was to evaluate RFQ concentrations in joint synovial fluids and bone tissues, plasma pharmacokinetics (PK), safety, and tolerability of RFQ for injection in adult patients undergoing total hip or knee arthroplasty (THA/TKA).

**Figure:**
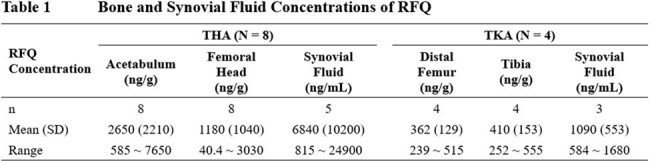

**Methods:**

This was a single-center, phase 1, open-label study (NCT04294862) conducted in the USA. Eligible patients received a single IV infusion of RFQ 300 mg 2 hours before anesthesia. Bone and synovial samples were collected during the arthroplasty surgery and analyzed for RFQ concentrations.

**Results:**

Between 1 Mar 2021 and 16 Dec 2021, 13 (9 THA and 4 TKA) patients were enrolled in the study. One subject did not receive a full dose of RFQ and was excluded from PK analysis. The mean bone RFQ concentrations were 2650, 1180, 362 and 410 ng/g in acetabulum, femoral head (THA patients), distal femur and tibia (TKA patients), respectively. The mean synovial fluid concentrations of RFQ were 6840 and 1090 ng/mL in THA and TKA patients, respectively. The mean plasma concentration-time profiles were comparable between THA and TKA patients and consistent with the PK profiles in previous studies. Four (30.8%) patients experienced at least one drug-related treatment-emergent adverse event (TEAE). There were no severe TEAEs or TEAEs leading to discontinuation of study drug.

**Conclusion:**

RFQ was safe and well tolerated in THA and TKA patients. The mean bone and synovial fluid concentrations of RFQ at about 2 hours after a single 300 mg infusion were all reached above the MBBC_90_ (250 ng/mL) level for *S. epidermidis*. The acetabulum and synovial fluid concentrations in THA patients also exceeded the MBBC_90_ (2000 ng/mL) level for *S. aureus*. The local concentrations of RFQ are expected to rise further and exceed the MBBC_90_ when patients are treated with multiple doses of RFQ.

**Disclosures:**

**Javad Parvizi, MD, FRCS**, TenNor Therapeutics (Suzhou) Ltd: Investigator **Chad A Krueger, MD**, TenNor Therapeutics (Suzhou) Ltd: Investigator **Jing Chen, Master of Science**, TenNor Therapeutics (Suzhou) Ltd: Employee **Huan Wang, PhD**, TenNor Therapeutics (Suzhou) Ltd: Employee **Changlin Ai, Master of Medicine**, TenNor Therapeutics (Suzhou) Ltd: Employee **Guozhu Geng, MD**, TenNor Therapeutics (Suzhou) Ltd: Employee **Zhenkun Ma, PhD**, TenNor Therapeutics (Suzhou) Ltd: Employee

